# Molecular Bases of Heat Stress Responses in Vegetable Crops With Focusing on Heat Shock Factors and Heat Shock Proteins

**DOI:** 10.3389/fpls.2022.837152

**Published:** 2022-04-11

**Authors:** Yeeun Kang, Kwanuk Lee, Ken Hoshikawa, Myeongyong Kang, Seonghoe Jang

**Affiliations:** ^1^World Vegetable Center Korea Office, Wanju-gun, South Korea; ^2^National Institute of Horticultural and Herbal Science (NIHHS), Rural Development Administration (RDA), Wanju-gun, South Korea; ^3^Biological Resources and Post-harvest Division, Japan International Research Center for Agricultural Sciences (JIRCAS), Tsukuba, Japan

**Keywords:** global warming, heat shock factor, heat shock protein, heat stress, thermotolerance, vegetables

## Abstract

The effects of the climate change including an increase in the average global temperatures, and abnormal weather events such as frequent and severe heatwaves are emerging as a worldwide ecological concern due to their impacts on plant vegetation and crop productivity. In this review, the molecular processes of plants in response to heat stress—from the sensing of heat stress, the subsequent molecular cascades associated with the activation of heat shock factors and their primary targets (heat shock proteins), to the cellular responses—have been summarized with an emphasis on the classification and functions of heat shock proteins. Vegetables contain many essential vitamins, minerals, antioxidants, and fibers that provide many critical health benefits to humans. The adverse effects of heat stress on vegetable growth can be alleviated by developing vegetable crops with enhanced thermotolerance with the aid of various genetic tools. To achieve this goal, a solid understanding of the molecular and/or cellular mechanisms underlying various responses of vegetables to high temperature is imperative. Therefore, efforts to identify heat stress-responsive genes including those that code for heat shock factors and heat shock proteins, their functional roles in vegetable crops, and also their application to developing vegetables tolerant to heat stress are discussed.

## Introduction

Vegetable crops mainly comprise sessile organisms. They routinely experience detrimental conditions including biotic and abiotic stresses in natural fields. The current climate changes including frequent extreme temperatures, strong storms, heavy rainfall, and harsh droughts directly threaten normal vegetable development during the entire period of vegetative and reproductive growth (Driedonks et al., 2016; Hansen et al., 2016; Bhutia et al., 2018). Global warming is one of the main issues related to global climate change and is caused by increases of greenhouse gases such as CO_2_, CH_4_, N_2_O, and hydrofluorocarbons (HFCs) that have been produced by urbanization and industrialization (Bhutia et al., 2018; Zandalinas et al., 2021). According to climate models (Driedonks et al., 2016) and the report from the Intergovernmental Panel on Climate Change (IPCC^1^), the world mean temperature will rise by 0.5 to 4°C in the twenty-first century (Hansen et al., 2016; Zandalinas et al., 2021). The changes in weather/climatic events such as temperature and rainfall are found to reduce the yield of crops. Statistical evidence shows that the temperature affects rice production in Africa. It was also found that irrigated rice yields in West Africa in the dry season would decrease by ∼45% due to reduced photosynthesis at extremely high temperatures (van Oort and Zwart, 2018). This indicates that the elevated temperature brought by climate change will result in significant losses in crop yields and production (Ortiz et al., 2008; Hansen et al., 2016). Plants have evolved to acquire the ability to induce defense mechanisms against the adverse effects of high ambient temperature on their growth (Ahuja et al., 2010; Bourgine and Guihur, 2021; Tian et al., 2021). The tolerance of plants to high ambient temperatures with no prior heat experience is known as basal thermotolerance (BTT), whereas the ability to overcome extremely high temperatures (HT) with pre-exposure to mild HT (i.e., sub-lethal temperatures) is known as acquired thermotolerance (ATT) (Ahuja et al., 2010; Bourgine and Guihur, 2021; Tian et al., 2021). The defense mechanisms against elevated temperatures in plants are tightly associated with rapid changes in gene expression in both BTT and ATT (Morimoto, 1998; Feder and Hofmann, 1999). Indeed, high ambient temperatures trigger a drastic cellular remodeling at the physiological and molecular levels in plants to maintain homeostasis, thereby allowing them to survive under adverse HT (Wang et al., 2004; Ohama et al., 2017; Tian et al., 2021). Within these mechanisms, how plants recognize HT and relay HT-induced signaling downstream to modulate transcription is a central question that plant researchers have been pondering for a long time. It has recently been reported that Ca^2+^ plays important roles in the perception, response, and adaptation of plants to heat stress (HS) (Mittler et al., 2012; Ohama et al., 2017; Lee and Seo, 2021). The alteration of fluidity in the plasma membrane (PM) in plants in response to HS can open cyclic nucleotide-gated calcium channels (CNGCs) controlled by nucleotide cyclases, thereby having Ca^2+^ move into the cytosol from the PM (Saidi et al., 2009; Finka et al., 2012; Gao et al., 2012; Mittler et al., 2012; Ohama et al., 2017). The Ca^2+^ ions are associated with protein calmodulin 3 (CaM3) during HS and the complex of Ca^2+^-CaM3 interacts with calcium/calmodulin-binding protein kinase 3 (CBK3) and phosphatase PP7 to transduce cytosol heat-stress response (HSR) signals into the nucleus by modulating phosphorytion and dephosphorylation of HSFA1, respectively (Liu et al., 2007, 2008; Mittler et al., 2012; Ohama et al., 2017). Also, the increased levels of Inositol-1,4,5-triphosphate (IP_3_) via the phosophoinositide-signaling pathway result in the influx of Ca^2+^ into cytoplasm from intracellular Ca^2+^ pools such as the endoplasmic reticulum (ER) and vacuole during HS (Zhang et al., 2009; Zhou et al., 2009; Mittler et al., 2012; Ohama et al., 2017). In addition, reactive oxygen species (ROS) produced by respiratory burst oxidase homolog B (RbohB), RbohD, and NADPH oxidases are other candidate sensors of HS (Königshofer et al., 2008; Miller et al., 2009; Suzuki et al., 2012). It has also been demonstrated that the ROS causes accumulation of nitric oxide (NO), which induces the activation of CaM3. The signaling cascade of CaM3 ultimately influences the association of DNA and heat shock factors (HSFs) in nucleus via the potential involvement of HSFA1 activity (Xuan et al., 2010; Wang et al., 2014; Ohama et al., 2017). Although Ca^2+^ and ROS are evaluated as predicted signal transducers during HS, the full activation of HSR in response of plants to HT cannot be exclusively explained by them. This indicates that there may be other signal transducers and multiple layers of signaling pathways including salicylic acid (SA), ethylene (ET), abscisic acid (ABA), and jasmonic acid (JA) signals (Fujita et al., 2006; Frank et al., 2009; Zhou et al., 2009).

The effect of HS on plants leads to diverse changes in plant cells including the state of cellular membranes, structural alterations in DNA and RNA species, and conformational changes of proteins, cytoskeleton structures, and metabolites (Ruelland and Zachowski, 2010; Mittler et al., 2012). For instance, high ambient temperature influences fluidity of cellular membranes containing primarily phospholipids, proteins, and carbohydrates with the modification of membrane rigidification (Ruelland and Zachowski, 2010). Also, high ambient temperature affects the accessibility of nucleic acids, and it has been determined that elevated temperatures induce the dissociation of the histone protein H2A.Z from nucleosomes, which promotes the chromatin accessibility to RNA polymerase II for the expression of genes for heat-shock proteins (HSP) and HSF, thus showing highly inductive and responsive gene expression dynamics (Kumar and Wigge, 2010; Zhang H. et al., 2021). RNA secondary structures can be affected by HS. It has been revealed that HT leads to a change in translation rate, resulting from the altered association of mRNAs with ribosomes (Matsuura et al., 2010). Since structured nucleic acid molecules melt as the temperature increases, it can be easily conceived that temperature changes affect the conformation of regulatory RNAs (Narberhaus et al., 2006). Indeed, the RNA secondary structure of internal ribosome entry sites (IRESs), which are translation regulatory elements of mRNAs, can be modified by HS to initiate translation in a cap-independent manner (Dinkova et al., 2005; Ruelland and Zachowski, 2010). Conversely, RNA secondary structures that mask ribosomal binding sites at optimal temperature can be modified by HS, allowing the conversion of non-functional RNA to the competent RNA species with ribosomal recruitment (Narberhaus et al., 2006). Heat stress also influences the conformational changes of proteins that act as signaling effectors in response to HT in plants (Ruelland and Zachowski, 2010). In *Arabidopsis*, the oligomerization of thioredoxin and/or thioredoxin-like proteins is induced by HS, causing concomitant functional switching from a disulfide reductase and foldase chaperone to a holdase chaperone (Lee et al., 2009; Park et al., 2009). It has also been reported that the elevated temperatures from 27 to 42°C in tobacco, and from 20 to 42°C in *Arabidopsis* cause severe damage to cytoskeletones including microtubules (Smertenko et al., 1997; Müller et al., 2007). Furthermore, tobacco BY-2 cells exposed to heat (50°C, for 5 min) exhibited depolymerization of actin microfilaments (Malerba et al., 2010), and such a defective phenotype was also observed in *Arabidopsis* roots (Müller et al., 2007). Based on a report demonstrating that heat triggers the accumulation of HSP70 and the heat-activated MAP kinase (HAMK), both HSP70 and HAMK are likely to be necessary to disassemble the cytoskeleton under HS (Suri and Dhindsa, 2008). Altered enzymatic activities such as the catalytic rate, and the un- or mis-folding of enzymes can also be affected by HS, resulting in the imbalance of cellular metabolism in plants (McClung and Davis, 2010; Ruelland and Zachowski, 2010; Suzuki et al., 2012). The steady-state efflux and influx of metabolites such as sucrose, prolines, glycine-betaine, ascorbate, glutathione, and ROS play an important role in heat response and tolerance (Wang et al., 2004; Al-Whaibi, 2011; Mittler et al., 2012). Reactive oxygen species were initially regarded as a toxic by-product of aerobic metabolism. However, it is now apparent that ROS such as superoxide and hydrogen peroxide are able to function as signal molecules to induce the HSR (Miller et al., 2007, 2009; McClung and Davis, 2010; Ruelland and Zachowski, 2010; Suzuki et al., 2012). In particular, the levels of ROS are influenced by the participation of ROS-generating enzymes in plant response to HT (Königshofer et al., 2008). The acquisition of plant heat tolerance is closely associated with the synthesis of chaperone proteins and the levels of non-enzymatic antioxidants in response to HT (Kotak et al., 2007; Wahid et al., 2007; Frank et al., 2009; Rampino et al., 2009). Many reports have been published showing that HS influences protein conformation which can drive a protein to be denatured, aggregated, and un- or mis-folded, thereby being directly recognized by several HSPs (Yamada et al., 2007; Scharf et al., 2012; Ohama et al., 2017). Notably, plant HSPs play a crucial role in conferring plant tolerance to HS, and they help facilitate proper folding of target proteins by hindering denaturation and aggregation of the proteins as molecular chaperones (Ahuja et al., 2010; Jacob et al., 2017). For instance, under normal temperature conditions, HSFs regulate the HSR and form inactive multiprotein complexes with HSPs. On the other hand, under HS, HSFs dissociate from the complex and form phosphorylated trimers, thereby allowing their nuclear translocation and binding to heat-shock element (HSE) to induce transcription of target genes (Kotak et al., 2007; Ahuja et al., 2010; Scharf et al., 2012; Jacob et al., 2017; Ohama et al., 2017). Indeed, transcriptomic and proteomic analyses revealed that the abrupt changes in gene expression in response to high ambient temperatures enhance a selected regulatory response and synthesis of proteins linked to HSPs, HSFs, and HSR (Al-Whaibi, 2011; Jacob et al., 2017; Zandalinas et al., 2021). However, the players and their mode of action in heat perception, HS-signaling pathways and HSR still remain elusive in vegetable crops.

In this review, we give an overview of the HSPs with focus on vegetable crops. Heat shock proteins play an essential role in the regulation of HSFs and subsequently, the expression of heat responsive genes. Moreover, a better understanding of HSPs will enable us to widen our knowledge of interconnected mechanisms underlying the complex regulatory networks of HSFs and heat responsive genes at the physiological and molecular levels during the adaptation of plants against HS. We also discuss the potential applications of biotechnology for efficient development of crops with enhanced thermotolerance to cope with climate change.

## Heat Shock Proteins Involved in Heat Stress

**FIGURE 1:**
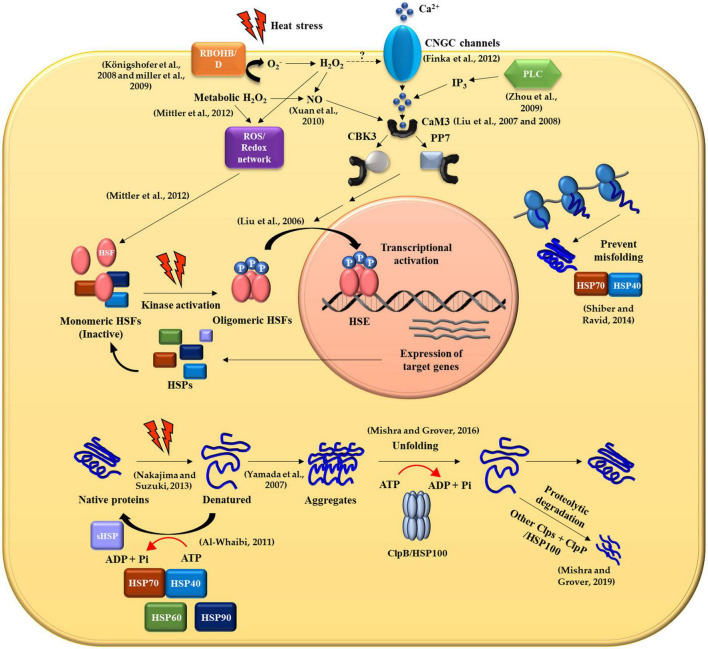
General molecular mechanism of heat shock protein production and transcriptional regulation in response to heat stress in plant cells.

**FIGURE 2:**
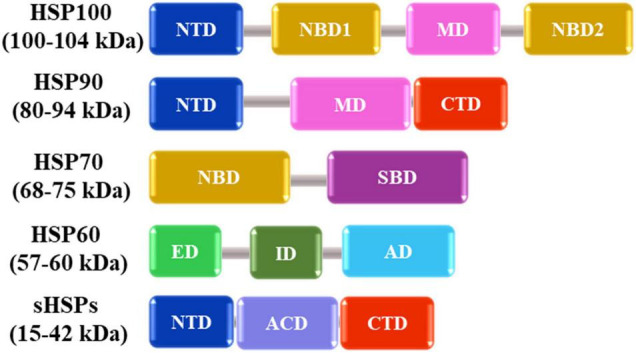
Schematic representation of available domains in the five major families of heat shock proteins. NTD (N-terminal domain), NBD (Nucleotide binding domain), MD (Middle domain), CTD (C-terminal domain), SBD (Substrate binding domain), ED (Equatorial domain), ID (Intermediate domain), AD (Apical domain), and ACD (Alpha-crystallin domain) are shown as boxes with different colors based on their functions. Numbers in parenthesis indicate the molecular weight distribution of each HSP family.

In nature, plants are often exposed to various kinds of abiotic stresses including low or high temperature, deficiency or excess of water, high salinity, heavy metals and ultraviolet radiation (Rucińiska-Sobkowiak, 2010; Bita and Gerats, 2013; Osakabe et al., 2013; He et al., 2018). Among these, HS has significant effects on plant growth, metabolism, and productivity (Rodríguez et al., 2015). HS causes protein misfolding and/or denaturation, leading to protein aggregation in plant cells by interactions between exposed hydrophobic amino acid residues of affected proteins (Nakajima and Suzuki, 2013). In response to HS, plants synthesize molecular chaperones including HSPs that recognize hydrophobic amino acid residues of non-native proteins and promote folding and refolding of denatured proteins (Figure 1). They are also responsible for assembling of multi-protein complexes, transporting, and sorting of proteins into correct compartments, controlling cell cycle and signal-transduction under various stress conditions. The different classes of HSPs play complementary and sometimes overlapping roles in protein stabilization under thermal stress. The HSPs are generally grouped into five major families based on their molecular weight: HSP100, 90, 70, 60 and the small HSPs (sHSPs) (Figure 2 and Table 1).

**TABLE 1:** Five major families of heat shock proteins and their major function under heat stress conditions.

HSP family/MW (kDa)	Subcellular location	Major functions under heat stress conditions	Major domain
HSP100/100-104	Cytosol Mitochondria Chloroplasts	Disaggregation of proteins and involvement in protein degradation (Mishra and Grover, 2016).	NTD (N-terminal domain) NBD (Nucleotide binding domain) MD (Middle domain)
HSP90/80-94	Cytosol ER Nucleus Mitochondria Chloroplasts	Protein folding, signal transduction (most of the substrates of HSP90s are kinases and transcription factors) (Kadota and Shirasu, 2012).	NTD MD CTD (C-terminal domain)
HSP70/68-75	Cytosol ER Mitochondria Chloroplasts	Assisting folding and refolding of non-native proteins to block protein degradation in the ER and protein import and translocation (Shiber and Ravid, 2014).	NBD SBD (Substrate binding domain)
HSP60/57-60	Mitochondria Cytosol ER Nucleus Chloroplasts	Assisting folding and refolding of unfolded polypeptides in the mitochondrial matrix (Martin et al., 1992; Caruso Bavisotto et al., 2020).	ED (Equatorial domain) AD (Apical domain) ID (Intermediate domain)
sHSPs/15-42	Cytosol ER Mitochondria Chloroplasts Membrane	Preventing aggregation and refolding of unfolded polypeptides (Waters and Vierling, 2020).	NTD ACD (Alpha-crystallin domain) CTD

Heat stress (HS) influences the alteration of membrane fluidity in plasma membrane (PM) *in planta* and activates the cyclic nucleotide-gated calcium channels (CNGCs), resulting in the movement of Ca^2+^ into the cytoplasm from the apoplastic space. The Ca^2+^ ions are associated with protein calmodulin 3 (CaM3) during HS and the Ca^2+^-CaM3 complex binds to either calcium/calmodulin-binding protein kinase 3 (CBK3) or phosphatase PP7 to transduce cytosol heat-stress response (HSR) signals into the nucleus by modulating phosphorylation and dephosphorylation of the heat shock transcription factors (HSFs), respectively. The elevated levels of inositol-1,4,5-triphosphate (IP_3_) via the phosophoinositide-signaling pathway (PLC) lead to an influx of Ca^2+^ into the cytoplasm from the pool of intracellular Ca^2+^ ions including the ER and vacuoles in response to HS and induce the same CaM3 signaling pathway. ROS are generated by respiratory burst oxidase homolog B (RbohB) and D (RbohD) during HS. RbohB/D-produced O_2_^–^ is converted into H_2_O_2_, which depolarizes PM as well as inducing the ROS/Redox signaling network which is involved in the activation of HSFs. Also, H_2_O_2_ is possibly increased in plant cells due to metabolic imbalances and the production of ROS, resulting in the accumulation of nitric oxide (NO) and the activation of calcium-channels that subsequently trigger the activity of CaM3 as illustrated in the (**Figure 1**). Upon HS stimuli, HSP interacts with unfolded and aggregated proteins, thereby releasing HSF monomer. Heat shock factor monomers trimerize and bind to HSEs within promoter regions of heat shock genes. Heat shock factors undergo several post transcriptional modifications (PTMs) such as phosphorylation, which regulate the transactivation capacity of HSF. Under normal conditions, HSPs directly bind to HSF and provide negative feedback required to deactivate HSF. HSP70 and HSP40 together function as ATP-driven machines that prevent aggregation of misfolded polypeptides and participate in protein refolding. When denatured or misfolded proteins form aggregates, ClpB/HSP100 is crucial for protein disaggregation, refolding or degradation by protease especially during HS. Consequently, HSPs as chaperones play a pivotal role in conferring thermotolerance in plants. The dashed line indicates an unknown pathway.

### Heat Shock Protein 100 Family

The caseinolytic proteinase/heat shock protein 100 (Clp/HSP100) proteins are members of the AAA+ protein group (ATPases associated with various cellular activities) that act in protein disassembly and/or protein degradation using the energy from adenosine triphosphate (ATP) hydrolysis (Sauer et al., 2004; Burton and Baker, 2005; Gul et al., 2021). In contrast to the typical molecular chaperones which function in protecting proteins from misfolding and aggregation, the Clp/Hsp100 proteins play a wide variety of functional roles in eliminating non-functional proteins and/or assisting the reassembly of denatured proteins from the aggregated protein complexes. As such, the Clp/Hsp100 proteins contribute to the maintenance of protein homeostasis in cells (Schirmer et al., 1996; Latterich and Patel, 1998; Agarwal et al., 2001; Mishra and Grover, 2019). The Clp/Hsp100 proteins consist of hexameric rings and the structural features are determined by nucleotide binding domains (NBD), spacer (linker) region, the middle domain (MD), N-terminal domain (NTD) and C-terminal domain (CTD) among diverse living organisms from prokaryotes to eukaryotes (Dougan et al., 2003; Schlieker et al., 2005; Butler et al., 2006). On the basis of the number of NBD domains, the Clp/Hsp100 family is classified into two major subclasses (class I and class II). The first class ClpA, ClpB, ClpC, and ClpD proteins that harbor two nucleotide binding domains (called ATP-binding domains) separated by spacers are clustered as large Clp proteins ranging from molecular weights of 68 to 110 kDa (Wang et al., 2004), whereas the second class including ClpM, ClpN, ClpX, and ClpY proteins that possess one NBD are grouped based on their low molecular weights ranging from 40 to 50 kDa (Wang et al., 2004; Mogk et al., 2008; Mishra and Grover, 2016). It was initially reported that the system of Clp ATPase proteins are able to hydrolyze casein *in vitro* (Hwang et al., 1987; Katayama-Fujimura et al., 1987). Later, further investigations on two-component protease systems revealed that the complexes of ClpA regulatory machine with an AAA+ ATPase module and a proteolytic component ClpP (Schelin et al., 2002) together with Lon protease complex serve as protein choppers for the degradation of toxic protein aggregates in cells (Wang et al., 2007). Moreover, the ClpAP complex recognizes target aggregated proteins via the guidance of the ClpS adapter that assists ClpAP to specifically bind and chop the aggregated proteins (Dougan et al., 2002). In addition to this, ClpB was initially found in bacteria and yeast, and it was later reported that plant HSPs were identified with high molecular weights of 100–110 kDa (Schirmer et al., 1994). Since plants harbor semi-autonomous organelles such as chloroplasts and mitochondria, plant ClpBs are classified into three different forms ClpB-C (cytoplasmic), ClpB-P (chloroplastic), and ClpB-M (mitochondrial) (Mishra and Grover, 2014). Although ClpB is considered to be a functional ortholog of ClpA with high similarity between the two proteins (Gottesman et al., 1990; Sanchez and Lindquist, 1990), it has been experimentally shown that ClpB could not replace the function of ClpA in protein degradation due to the lack of the LIV-GFL motif required for the interaction with ClpP (Weibezahn et al., 2004; Zolkiewski, 2006; Tessarz et al., 2008). Moreover, it was demonstrated that ClpB plays an essential role in the denaturing and/or renaturing pathway to release the native proteins from the aggregates rather than the degradation pathway as other Clps do. Of note, it has been displayed that ClpB is induced by HS in contrast to other Clps (Singh et al., 2010; Kim et al., 2012), indicating that ClpB is crucial for the protein renaturation/denaturation from aggregates especially during HS. Interestingly, the possible mechanism for assisting protein folding toward native and functional form from aggregates would be collaborated with the Hsp70 member, which is another ATP-dependent chaperone that is involved in refolding of liberated proteins by ClpB/HSP100 (Glover and Lindquist, 1998; Goloubinoff et al., 1999). However, when the aggregated proteins are interacted with other Clps and the peptidase (ClpP) system, the proteins move to the degradation pathway (Wang et al., 2004). The cellular roles of ClpB have been widely studied from prokaryotes to eukaryotes such as bacteria, yeast, and plants (Lindquist, 1986; Vierling, 1991; Wang et al., 2004). Remarkably, it has been determined that the fine-tuned expression of ClpB genes within cells is required for normal growth, development, and adaptation to environmental stresses including cold, heat, drought, and high salt (Yang et al., 2006). In particular, it has been shown that ClpB proteins are essential for rendering thermotolerance to organisms in response to HS. The loss-of-function mutant of *ClpB* in *E. coli* remarkably affected cell viability in response to abrupt HT (50 °C) with a slow growth rate at 44 °C (Squires et al., 1991). Also, *ScHSP104* in *Saccharomyces cerevisiae* is one of the *ClpB* genes involved in acquiring thermotolerance: *ScHSP104* deficient yeast cells grew and died at the same rate as the wild-type cells did when exposed directly to HT although the mutant cells could not acquire tolerance to heat after a mild pre-heat treatment (Sanchez and Lindquist, 1990). Plant ClpB/HSP100 proteins have been evaluated in diverse plant species including *Arabidopsis* (Lee et al., 2007), wheat (Campbell et al., 2001), soybean (Lee et al., 1994), maize (Nieto-Sotelo et al., 1999; Young et al., 2001), and rice (Agarwal et al., 2003). Analyses of ClpB/HSP100 proteins have been also conducted in vegetable crops such as pea, tomato, pepper, carrot, spinach, potato, banana, rapeseed, and mustard greens in response to heat and cold stresses.

### Heat Shock Protein 90 Family

Heat shock protein 90 (HSP90; known as GroEL in *E*. *coli*) is one of the most abundant heat-related proteins expressed in cells accounting for 1–2% of total protein levels (Taipale et al., 2010). Heat shock protein 90 is a highly conserved molecular chaperone involved in the assembly, maturation, stabilization and activation of key signaling proteins including regulatory kinases, steroid hormone receptors and transcription factors in plant cells (Kadota and Shirasu, 2012; Chen et al., 2019). Most plants have several isoforms of HSP90 classified by their subcellular localization in the cytoplasm (HSP90.1), nucleus (HSP90.4), chloroplast (HSP90.5), mitochondria (HSP90.6), and endoplasmic reticulum (ER; HSP90.7) (Milioni and Hatzopoulos, 1997; Krishna and Gloor, 2001; Xu et al., 2012). HSP90 exists in the form of a dimer consisting of three main structural domains: NTD, which binds ATP; MD, which is important for ATP hydrolysis and client protein binding; and CTD, which mediates HSP90 dimerization and client protein binding. ATP binding to the NTD and its hydrolysis induce conformational change which is essential for chaperone activity (Krishna and Gloor, 2001; Pearl and Prodromou, 2006). HSP90 proteins play a major role in assisting the proper folding of other proteins together with HSP70s (Picard, 2002) by acting as molecular chaperones, signaling for the cellular quality control, trafficking of other HSP proteins (Pratt and Toft, 2003) and stabilizing proteins against HS (Marcu et al., 2002; Wang R. et al., 2016). Also, HSP90 proteins along with their co-chaperone HSP70s contribute to the maintenance of cellular protein homeostasis by inactivating HSF during attenuation/recovery of HSR (Hahn et al., 2011). In *Arabidopsis*, HSP90 and the co-chaperone SUPPRESSOR OF G2 ALLELE SKP1 (SGT1) positively regulate plant growth by stabilizing the auxin co-receptor F-box protein TIR1 under higher ambient temperature conditions (Wang R. et al., 2016), showing that HSP90 participates in plant growth control under changing thermal conditions.

### Heat Shock Protein 70 Family

The heat shock protein 70 (HSP70) family (known as DnaK in *E. coli*), one of the most ubiquitous classes of chaperones, is highly conserved in all organisms, and also found in different cellular compartments such as the cytosol, chloroplasts, ER and mitochondria (Amir-Shapira et al., 1990; Radons, 2016; Usman et al., 2017). The HSP70 family is the central hub of the protein homeostasis network that prevents protein aggregation and uses the energy of ATP hydrolysis to solubilize, translocate and mediate the proper refolding and unfolding of proteins (Ben-Zvi et al., 2004; Imamoglu et al., 2020). Heat shock protein 70 contains two major domains: one is the N-terminal nucleotide binding domain for hydrolyzing ATP to ADP (Adenosine diphosphate) and the other is the C-terminal substrate binding domain (SBD) (Mayer, 2010). Under abiotic stress conditions such as HS, HSP70 molecular chaperones also function as ATP-driven unfolding/refolding machines that are capable of shifting substrate polypeptides between various folding states together with their co-chaperones such as HSP40 (Lee et al., 2007; Shiber and Ravid, 2014; Palakolanu et al., 2016). The significance of HSP70 regarding functional roles against HS was highlighted by transgenic plants overexpressing *AtHSP70-1* and *NtHSP70-1* (Sung and Guy, 2003; Cazalé et al., 2009; Cho and Choi, 2009). In addition, numerous experimental results have shown that HSP70 is involved in thermotolerance in various crops such as rice (Jung et al., 2013), tomato (Hahn et al., 2011), and pepper (Guo et al., 2014) under HS conditions.

### Heat Shock Protein 60 Family

The heat shock protein 60 (HSP60) family (also known as chaperonins, Cpn, and GroEL in *E. coli*) typically functions inside the mitochondria together with the co-chaperone HSP10 to maintain protein homeostasis (Caruso Bavisotto et al., 2020). However, they have also been found in other subcellular compartments including the ER, cytosol, chloroplasts and nucleus, and participate in folding and aggregation of many proteins (Meng et al., 2018). Chaperonins are generally composed of two rings, stacked back to back, consisting of subunits of ∼60 kDa molecular weight (Nguyen et al., 2021). Each oligomer has three domains (1) the equatorial domain (ED), which has the ATP-biding site, (2) the apical domain (AD), which hosts client proteins and (3) the intermediate domain (ID), which transduces signals from the equatorial domain (Pipaón et al., 2021). When signals are transmitted to the ID from ATP binding and hydrolysis, conformational changes occur in the AD corresponding to the open and closed forms (Xu et al., 1997). Heat shock protein 60 proteins bind several types of proteins before folding to block their aggregation (Parsell and Lindquist, 1993) and stromal chaperones (Hsp70 and Hsp60) are involved in functional conformation of newly transferred proteins to the chloroplast (Jackson-Constan et al., 2001). Most of the HSP60 family proteins are heat inducible and also required for preventing protein aggregation, and mediating folding and refolding in mitochondria under HS conditions (Martin et al., 1992; Sharma et al., 2006).

### Small Heat Shock Protein Family

Small heat shock proteins (sHSPs), which have a low molecular mass of 15-42 kDa, are very diverse in plants (Wang et al., 2004; Basha et al., 2006; Morrow and Tanguay, 2012). Small heat shock proteins have a common alpha-crystallin domain (ACD) containing 80–100 amino acid residues on the C-terminal region, and contribute to degradation of proteins with unsuitable folding (Seo et al., 2006). Small heat shock proteins are ubiquitous ATP-independent molecular chaperones that bind and stabilize misfolded or unfolding intermediates of substrate proteins in an energy-independent manner (Ferguson et al., 1990; Miernyk, 1999; Waters and Vierling, 2020).

## Transcriptional Regulation of Heat Shock Proteins in Plants Under Heat Stress

Heat-stress response is known to be controlled by complex, tight networks, including selective enhancement and repression of gene expression in various metabolic processes, production of chaperone proteins for cellular protein homeostasis and other protective molecules that prevent targets from detrimental effectors such as ROS. The regulation of this network is critical for plant cells not only to adapt to various environmental conditions linked to temperature, humidity and light, but also to protect them from proteotoxic stresses. HSFs have a central function as major regulators in HSR by regulating transcription of a wide range of genes in several signaling and metabolic pathways (von Koskull-Döring et al., 2007; Guy et al., 2008). Heat shock factors are responsible for rapid synthesis and accumulation of HSPs, molecular chaperones for preventing protein aggregation and maintaining cellular protein homeostasis (Vierling, 1991; Wang et al., 2004; Gupta et al., 2010; Schleiff and Becker, 2011). Heat shock factor activity in each cell is controlled through sophisticated and complex feedback mechanisms and protein interactions, allowing for rapid adjustment and flexibility by diverse chaperones to changing environmental conditions (Akerfelt et al., 2010).

The expression of *HSPs* is induced by HSFs that bind the HSEs in the promoters of heat shock responsive genes (Nover et al., 2001). Under normal conditions, monomeric HSFs are bound to HSP70 in the cytoplasm. When plants are exposed to HS, HSFs are released from HSP70-HSF complexes, and phosphorylated in the cytoplasm, and form a trimer for biding to HSEs in the nucleus (Liu et al., 2006). Overexpression of HSF genes in turn turns on almost all heat shock genes containing the HSE consensus sequence, conferring tolerance to HS. HSP70/90 plays an important role in the regulation of HSFA1 activity. HSP70/90 complex keeps HSFA1 inactive under normal conditions by repressing transactivation activity and nuclear localization of HSFA1 (Yamada et al., 2007; Hahn et al., 2011). Recently, the temperature-dependent repression (TDR) domain has been identified in the central region of HSFA1d, one of the *Arabidopsis* HSFA1s responsible for HS-dependent transactivation activity (Ohama et al., 2017). Overexpression of constitutively active HSFA1d, which lacks the TDR domain, induced the expression of heat shock proteins in the absence of HS, thereby conferring strong thermal stability in the overexpressing plants. Under HS conditions, HSFA1a is released from the HSFA1-HSP70/90 complex and activated. Of note, no TDR domain has been observed in mammalian HSFA1 proteins although the repression of the activities of HSFs by the HSP70/90 complex is generally conserved in both plants and animals. Activated *HSFA1* directly and rapidly regulates expression levels of genes encoding important HS-responsive transcription factors (TFs) such as DEHYDRATION-RESPONSIVE ELEMENT BINDING PROTEIN 2A (DREB2A), HSFA2, HSFA7a, HSFBs, and MULTIPROTEIN BRIDGING FACTOR 1C (MBF1C) (Yoshida et al., 2011). Subsequently, DREB2A directly regulates the gene expression level of *HSFA3* by creating a coactivator complex with NUCLEAR FACTOR Y, SUBUNIT A2 (NF-YA2), NF-YB3, and DNA POLYMERASE II SUBUNIT B3-1 (DPB3-1)/NF-YC10 (Chen et al., 2010; Sato et al., 2014). *HSFA3* knockout or knockdown transgenic lines caused reduced expression of putative target *HSP* genes under HS, thus HSFA3 is regarded as an important HS-responsive TF (Schramm et al., 2008; Yoshida et al., 2008). Furthermore, HSFA2 contributes to high levels of modifications at specific histone tail residues (H3K4me2 and H3K4me3) of *ascorbate peroxidase 2* (*APX2*), *HSP22*, and *HSP18.2* (Sung et al., 2003; Charng et al., 2007; Lämke et al., 2016). Heat stress memory is maintained for several days, allowing plants to survive when they are exposed to the next HS conditions (Yamaguchi, 2021). Strong/rapid expression of *sHSP* genes including *HSP21*, *HSP22*, and *HSP17.6C* is observed in primed plants compared to non-primed plants (Yamaguchi et al., 2021). *FORGETTER3* (*FGT3*)/*HSFA3* is needed to retain HS memory for several days following HS exposure (Friedrich et al., 2021). A recent discovery showed that genes encoding stem cell regulators such as *CLAVATA1* (*CLV1*), *CLV3*, and *HSP17.6A*, and the primary carbohydrate metabolism gene *FRUCTOSE-BISPHOSPHATE ALDOLASE 6* (*FBA6*) are involved in the HS transcriptional memory in the shoot apical meristem (Olas et al., 2021). *JUMONJI-C DOMAIN CONTAINING PROTEINs* (*JMJs*) that code for H3K27me3 demethylases are regulators of heat acclimation through controlling the methylation status of *HSP* loci (Pan et al., 2007; Xiao et al., 2016; Yamaguchi et al., 2021; Yamaguchi and Ito, 2021).

Extreme HT causes protein misfolding and denaturation. Unfolded proteins can be degraded by the ubiquitin proteasome system or autophagy (Buchberger et al., 2010; Amm et al., 2014; Xu and Xue, 2019). It has been demonstrated that some ubiquitin E3 ligases and autophagy-related genes play a critical role in plant heat tolerance (Zhou et al., 2014; Li et al., 2015; Liu J. et al., 2016; Gil et al., 2017). Transgenic plants overexpressing ubiquitin or ubiquitin E3 ligases displayed enhanced BTT and/or ATT (Tian et al., 2014; Liu J. et al., 2016), and Zhang Y. et al. (2021) reported that silencing CARBOXYL TERMINUS OF THE HSC70-INTERACTING PROTEINS (CHIP), a chaperone-dependent ubiquitin E3 ligase caused reduced heat tolerance in tomato. CHIP plays a critical role in HSR through the misfolded proteins degradation induced by HS. Transgenic *Arabidopsis* seedlings overexpressing PROTEIN WITH THE RING DOMAIN AND TMEMB (PPRT1) encoding a C3HC4 zinc-finger ubiquitin E3 ligase showed enhanced BTT and ATT (Liu Y. et al., 2020). Moreover, virus-induced gene silencing (VIGS) of tomato *AUTOPHAGY RELATED5* (*ATG5*) and *ATG7* genes resulted in increased sensitivity of tomato plants to HS (Zhou et al., 2014).

Understanding the dynamic behavior involving expression levels of TFs and HSPs under HS will help understand the whole regulatory network to adapt to HT.

### Expression Patterns of *HSP* and *HSF* Genes in Vegetables Under Heat Stress

Exposure to extreme temperature stresses such as heat and cold induces cellular changes in plant cells (Guy, 1999; Bita and Gerats, 2013). Plants have evolved various physiological and molecular adaptations to stresses in order to minimize damage and provide cellular homeostasis (Theocharis et al., 2012; Awasthi et al., 2015). In response to the extreme temperature stresses, plants synthesize many stress-responsive proteins including HSP and HSF by regulating gene expression (Guo et al., 2016a; Ul Haq et al., 2019). So far, many studies on gene expression patterns under heat and/or cold stresses in vegetable crops have been reported and the collected information can be seen in Table 2.

**TABLE 2:** Gene expression pattern response to heat or cold stress in vegetables.

Vegetables	Gene/protein	Expression pattern		Tissue	Description	References
		Heat (H)	Cold (C)			
Tomato (*Solanum* *lycopersicum)*	*SlHSP100*	Up		H: leaves	Upregulation detected in both thermotolerant and thermosensitive lines under HS.	Gul et al., 2021
	HSP70 sHSP	Up	*Up (H → C)	H/C: fruits	Protein levels of HSPs were increased under HS. *Increased protein levels at HT remained high for several weeks even when transferred to low temperatures.	Sabehat et al., 1996
	*SlHSP20*	Up/Down			Expression of 13 of all tested *SlHsp20* genes was drastically increased in both thermotolerant and thermosensitive lines under HS, except for *SlHsp15.7.*	Yu et al., 2016
	*HSFA2* *Hsp17-CII*	Up		H: flowers	The highest induction of two genes was identified in the anther tissues under HS.	Giorno et al., 2010
	*tom111* (homolog from pea *HSP21*), *tom66*, (homolog from pea *HSP18.1*)	Up	**Up (H → C)	H: fruits, flowers, leaves, stems C: Mature-green fruits	The expression of *tom 111* and *tom66* was induced by HT. **The expression was first decreased and re-induced after the heated organs were transferred to low temperature.	Sabehat et al., 1998
	*LeHSP17.6*	Up	***Up (H → C)	H/C: fruits	Finally, Fruits with heating-and-chilling treatment showed a high level of expression of LeHSP17.6. ***Increased expression of *LeHSP17.6* at HT remained during subsequent exposure to low temperatures for at least one week.	Kadyrzhanova et al., 1998
Pepper (*Capsicum* *annuum*)	*CaHSP70*	Up/Down		H: leaves	Expression of *HSP70* gene was highly upregulated in the thermotolerant line compared to the thermosensitive line under HS.	Usman et al., 2015
	*CaHSP60*	Up/Down	Up	H/C: leaves, stems, roots	Fifteen (93% of total *CaHSP60* genes) *CaHSP60* genes were upregulated under HS and cold stress, and only *CaHSP60-3* was downregulated in both thermosensitive B6 and thermotolerant R9 lines.	Haq et al., 2019
	*CaHSP20*	Up/Down		H: leaves, stems, roots, flowers	Generally, the peaks of expression levels of *CaHsp20* genes in the thermosensitive line B6 were higher than the thermotolerant line R9.	Guo et al., 2015
	*CaHSP16.4*	Up		H: leaves, roots	The expression level of *CaHsp25.9* was higher in leaves than that in roots, and was highest at 2 h after HS in both thermosensitive B6 and thermotolerant R9 lines.	Feng et al., 2019
Soybean (*Glycine max)*	*GmHSP90*	Up		H: leaves	A significant upregulation was observed in 12. *GmHsp90* genes within 30 min at 42°C	Xu et al., 2013
	*GmHSP70*	Up/Down		H: leaves	29 genes out of 61 detectable *GmHSP70s* showed upregulation under drought and HS conditions.	Zhang et al., 2015
	*GmHSP20*	Up	Up	C: leaves	47 soybean *Hsp20* genes were responsive to heat shock stress, and 5 were also induced by cold stress.	Lopes-Caitar et al., 2013
Pea (*Pisum sativum*)	*HSP70* *PsHSFA*	Up		H: leaves, cotyledons	The expression of *PsHSFA* and *HSP70* was induced in both leaves and cotyledons under HS.	Aranda et al., 1999
	*HSP17.9* *HSP18.1*	Up		H: leaves	The expression of *HSP17.9* and *HSP18.1* was highly upregulated at the beginning of HS, and declined rapidly after the stress.	DeRocher et al., 1991
Potato (*Solanum* *tuberosum)*	18 kDa sHSP	Up		H: leaves	The 18 kDa sHSP proteins were induced longer in the heat tolerant cultivars than the heat sensitive cultivars.	Ahn et al., 2004
	HSP100 HSP90 HSP80 HSP70 sHSP		Up (during chilling storage)	C: tuber	Fifteen *HSPs* genes, including *HSP100*, *HSP90*, *HSP80*, *HSP70* and *sHSP* family were consistently upregulated by low temperatures in both RNA and protein levels, which may act to prevent cellular damage from cold stress in potato tubers during postharvest storage.	Lin et al., 2019
Lettuce (*Lactuca sativa*)	*HSP70*	Up		H: leaves, stems	HT induced the expression of a gene encoding HSP70 that interacts with a calmodulin for heat induced bolting tolerance.	Liu R. et al., 2020
	*HSP70* *sHSP*	Up		H: leaves	The *sHSP* and *HSP70* genes were quickly and sharply induced within 1 h treatment of HS.	Kang et al., 2021

### Tomato (*Solanum lycopersicum* L.)

Tomato is one of the most economically important vegetable crops worldwide (Campos et al., 2021). As global warming leads to extreme weather events, a number of researchers have examined the effects of heat and/or cold stresses on the expression pattern of genes such as *HSPs* and *HSFs*, which play crucial roles in thermotolerance in tomatoes (Tubiello et al., 2007).

Heat treatment has been found to induce chloroplastic *SlHSP100* genes in both thermotolerant and thermosensitive tomato seedlings. The highest upregulation was observed in the genotype 17903, which showed the highest ratio of cell viability and cell membrane stability under HS, implying a crucial role for the gene in ATT (Gul et al., 2021). Besides the role of HSP100 as a chaperone, Sabehat et al. (1996) found that tomato fruits heated and then chilled showed a high level expression of both *HSP70* and *sHSP* family genes (14–25kDa) and enhanced chilling tolerance compared to unheated fruits (Sabehat et al., 1996). Similar results were also reported by Kadyrzhanova et al. (1998) and Sabehat et al. (1998) where the expression of chloroplastic *HSP21* and *HSP17.6* was first decreased and re-induced when the heated fruits were transferred to low temperature. The members of *SlHSP20s* in tomato were also upregulated in both thermotolerant and thermosensitive lines under HS, except for *SlHsp15.7* (Yu et al., 2016). Moreover, it has been reported that the expression of *HSFA2*, transcriptional activator of *HSP* expression, and *HSP17-CII* was highly activated in the tomato anther during its development under HS (Giorno et al., 2010).

### Pepper (*Capsicum annuum*)

The production and consumption of pepper has steadily increased worldwide due to its nutritional benefits and spice, but it is thermosensitive (Crosby, 2008; Guo et al., 2014). As with tomato, there has been a growing body of research that explores the expression of *HSP* genes in pepper under temperature stress conditions. Many *HSPs* including *CaHSP70*, *CaHSP60*, *CaHSP20*, and *CaHSP16.4* are upregulated in pepper under HS (Guo et al., 2015; Usman et al., 2015; Feng et al., 2019; Haq et al., 2019). *HSP70* gene was significantly upregulated in the thermotolerant line compared to the thermosensitive line after 2 h of HS treatment at 42°C, indicating that the gene is quickly and sharply induced by heat shock and plays a major role in thermotolerance (Usman et al., 2015). Haq et al. (2019) observed that fifteen *CaHSP60* genes were upregulated under HS and cold stress, and only *CaHSP60-3* was downregulated in both thermosensitive B6 and thermotolerant R9 lines (Haq et al., 2019).

### Soybean (*Glycine max*)

Soybeans are members of the legume family of vegetables and have been a staple of Asian cuisines for a long time. Soybean yield is severely affected by temperature stresses. Under low or high temperature stress conditions, HSPs are induced in soybean to prevent cell damage caused by the temperature stresses. Xu et al. (2013) studied the expression of *GmHSP90* in relation to HS, and observed a significant upregulation of this gene in early response to HS (Xu et al., 2013). Expression patterns of soybean 61 *GmHSP70* genes under HS and drought were analyzed. Among those genes, 55 *GmHSP70* genes were highly upregulated during HS, and 29 *GmHSP70* genes showed increased expression under both heat and drought stress conditions, indicating that most of the *GmHSP70* genes play an important role in heat and drought tolerance (Zhang et al., 2015). Similarly, 47 *GmHSP20* genes among 51 *GmHSP20* candidates were found to be highly induced under HS and 5 genes were induced under both heat and cold conditions (Lopes-Caitar et al., 2013).

### Pea (*Pisum sativum*)

Pea has long been important in the human diet due to its starch, protein, and fiber content and the many phytochemical substances it contains, but it is a cool season crop which is heat-sensitive (Dahl et al., 2012). Therefore, some researchers have investigated the expression of *HSPs* in pea during HS. DeRocher et al. (1991) observed that the *HSP18.1* mRNA peaked at the beginning of the maximum temperature during 4 h gradual HS (30–42°C) period, and began to decline 6 to 8 h before the amount of HSP18.1 protein reached maximum levels, implying that sHSP levels in plants may also be self-regulated or regulated by some other heat-inducible protein.

### Potato (*Solanum tuberosum*)

Potato is a vegetable crop that mainly grows in a temperate climate, so HS can have a negative effect on the yield by inducing physiological defects in tubers (Rykaczewska, 2017). Hence, it is important to examine the accumulation of HSPs in response to HS. Ahn et al. (2004) reported that the 18 kDa sHSP proteins were synthesized for a longer time in the heat tolerant cultivars compared to the heat sensitive cultivars under strong heat shock temperature, suggesting that sHSP plays an important role in the heat tolerance enhancement (Ahn et al., 2004). Fifteen HSPs, including three HSP70s, two HSP80s, one HSP90, one HSP100 and eight sHSPs were consistently upregulated by low temperatures at both the RNA and protein levels to reduce cellular damage and re-build cellular homeostasis in potato tubers under cold stress during postharvest storage (Lin et al., 2019).

### Lettuce (*Lactuca sativa*)

Lettuce is an important cool season leafy vegetable with an optimal growing temperature ranging from 17 to 28°C (Holmes et al., 2019). HT can facilitate the accumulation of gibberellin (GA) which promotes lettuce bolding (Fukuda et al., 2012). Under HT, it is suggested that induced expression of genes encoding LsHSPs that interact with a calmodulin confers enhanced tolerance to heat with bolting resistance in lettuce (Liu R. et al., 2020). Recently, putative early heat responsive *HSP* genes were identified by transcriptome profiling in lettuce (Kang et al., 2021). Among them, *sHSP* and *HSP70* genes were quickly and sharply induced within 1 h in response to HS, indicating that these genes could be potential candidates as the breeding targets for the development of heat-tolerant lettuce cultivars.

## Breeding for Elevated Resistance to Heat Stress

Currently, the greatest risk to crop productivity and yields associated with global climate change is being caused by extreme weather events such as extreme hot and cold weather (Reddy and Hodges, 2000). Therefore, improved tolerance to heat and cold stress might be crucial in increasing yields for most crops. Application of transgenic and genome editing technologies could help to introduce desirable abiotic stress tolerance traits into crop varieties (Sanghera et al., 2011; Lamaoui et al., 2018). In recent years, there has been an increasing effort to reveal functional roles of *HSPs* and *HSFs* using mutagenic and transgenic plants for production of crops with enhanced heat and/or cold tolerance (Table 3).

**TABLE 3:** Engineering temperature stress tolerance in plants.

Transgenic plant	Stress	Gene targeted/transferred	Gene expression/manipulation	Result	References
*Arabidopsis*	Heat	*AtHSP101*	Down regulation/Antisense inhibition or co-suppression	Decreased heat tolerance.	Queitsch et al., 2000
		*AtHSF1*	Overexpression of *AtHSF1*-GUS and GUS-*AtHSF1*	Increased *HSP18* expression level at normal temperatures and enhanced basic thermotolerance.	Lee et al., 1995
		*CaHSP25.9* From pepper	Overexpression	Increased heat tolerance. Reduced accumulation of reactive oxygen species (ROS).	Feng et al., 2019
		*CaHSP70* from pepper	Overexpression	Increased heat tolerance including basal thermotolerance and acquired thermotolerance.	Guo et al., 2016b
		*PfHSP21.4* from Primula	Overexpression	Increased thermotolerance activity. Increased antioxidant enzymes such as ascorbate peroxidase (APX).	Zhang et al., 2014
		*TaHSP26* from wheat	Overexpression	Increased thermotolerance. Increased photosynthetic pigments, higher biomass, and seed yield.	Chauhan et al., 2012
			Down-regulation/Antisense inhibition	Showed negligible thermotolerance.	
		*LimHSP16.45* from David Lily	Overexpression of *LimHSP16.45*-GFP	Enhanced viability of *Arabidopsis* cells under HS. Induced more superoxide dismutase (SOD) and catalase (CAT) activity.	Mu et al., 2013
	Cold	*CsHSP17.7* *CsHSP18.1* *CsHSP21.8* from *Camellia sinensis*	Overexpression	Increased root length in *Arabidopsis* under low temperature.	Wang et al., 2017
		*PfHSP17.2* from Forrest primrose	Overexpression	Enhanced freezing tolerance.	Zhang L. et al., 2018
Tobacco	Heat	*OsHSP101* (*ClpB-C*) from rice	Overexpression	Increased heat tolerance.	Chang et al., 2007
		*ZmHSP16.9* from maize	Overexpression	Increased tolerance to heat and oxidative stress.	Sun et al., 2012
		*LeHSP21* from tomato	Overexpression	Increased tolerance to heat and oxidative stress.	Zhang et al., 2016
		*BcHSP70* from *Brassica campestris*	Overexpression	Increased heat tolerance. Increased the chlorophyll content, SOD and peroxidase (POD) activities.	Wang X. et al., 2016
	Cold	*CaHSP26* from sweet pepper	Overexpression	Protected PSII and PSI from chilling stress.	Guo et al., 2007
		*CaHSP22.5* from pepper	Overexpression	Improved the tolerance of chilling stress. Increased the activity of reactive oxygen species-scavenging enzymes.	Li et al., 2018
Rice	Heat	*AtHSP101* (*ClpB-C*)	Overexpression	Increased heat tolerance.	Katiyar-Agarwal et al., 2003
		*OsHSP18.6*	Overexpression	Increased heat tolerance. Exhibited the lower levels of malondialdehyde (MDA) and greater CAT and SOD activities.	Wang et al., 2015
Tomato	Heat	*HSFA1b* (*AtHSF* *A1b* and β*-glucuronidase* (*gusA*) fusion gene)	Overexpression	Increased heat tolerance. Increased the activity of soluble isoforms of APX.	Li et al., 2003
		*HSP24.4*	Overexpression	Increased heat tolerance. Showed tissue specific expression in root, shoot, and stem tissue under HS.	Mahesh et al., 2013
		Unknown (*HT7* mutant)	EMS Micro-Tom mutant	Heat tolerant tomato lines. Highly expressed *SlHSFA1b* and *SlHsp101* than WT respond to HS.	Pham et al., 2020
	Cold	*HSP*	Overexpression	Increased chilling tolerance.	Wang et al., 2005
		*HSFA1b* (*AtHSF* *A1b* and *gusA* fusion gene)	Overexpression	Increased chilling tolerance. Increased the activity of soluble isoforms of APX.	Li et al., 2003
		*sHSP23.8-M*	Overexpression	Protected fruit from chilling injury.	Escobar et al., 2021
			Knock-down	Decreased chilling tolerance. Showed wilting and skin wrinkles, partial discoloration.	
		*SlHSP17.7*	Overexpression	Increased tolerance response to cold stress.	Zhang et al., 2020
Potato	Heat	*DcHSP17.7* from carrot	Overexpression	Increased cellular membrane stability and tuberization.	Ahn and Zimmerman, 2006
Pepper	Heat	*CaHSP60-6*	Down regulation/virus-induced gene silencing (VIGS)	Reduced heat tolerance.	Haq et al., 2019
Carrot	Heat	*HSP17.7*	Overexpression	Increased heat tolerance (with an increase of 68-90% growth).	Malik et al., 1999
			Down-regulation/Antisense inhibition	Decreased heat tolerance (with a decrease of 12-26% growth).	
Soybean	Heat	*GmHsp90A2*	Overexpression	Increased heat tolerance. Reduced chlorophyll loss and stabilized membrane systems.	Huang et al., 2019
			Knockout/CRISPR/Cas9	Reduced heat tolerance.	

### Model Plants

A number of researchers have used model plants such as *Arabidopsis*, tobacco and rice for functional studies (proof of concept) on genes involved in heat and cold stresses because of the ease of genetic experiments (Rensink and Buell, 2004; Koornneef and Meinke, 2010). Queitsch et al. (2000) examined transgenic *Arabidopsis* plants containing *HSP101* antisense and/or co-suppression constructs, and found that they showed normal growth but impaired ATT and BTT, indicating *HSP101* plays a pivotal role in heat tolerance in *Arabidopsis*. In contrast, transgenic *Arabidopsis* plants containing constitutively active HSF-GUS fusion proteins caused increased *HSP18* expression at normal temperature by forming HSF trimers and their binding to DNA, resulting in enhanced BTT (Lee et al., 1995).

In addition, transgenic approaches with other crop genes have also been made with a fair degree of success. Genetically engineered *Arabidopsis* plants overexpressing *HSP* genes from pepper (Guo et al., 2016b; Feng et al., 2019), primula (Zhang et al., 2014), wheat (Feng et al., 2019) and David Lily (Mu et al., 2013) exhibited increased thermotolerance activity. Similar events were also observed under cold stress conditions by Wang et al. (2017) and Zhang L. et al. (2018). They introduced *CsHSP17.7*, *CsHSP18.1*, *CsHSP21.8*, and *PfHSP17.2* from *Camellia sinensis* and Forrest primrose into *Arabidopsis* for overexpression. Transgenic plants showed increased root length and tolerance to cold stress. Furthermore, overexpression of *OsHSP101* (Chang et al., 2007), *ZmHSP16.9* (Sun et al., 2012), *LeHSP21* (Zhang et al., 2016), *BcHSP70* (Wang X. et al., 2016), *AtHSP101* (Katiyar-Agarwal et al., 2003) and *OsHSP18.6* (Wang et al., 2015) conferred improved HS tolerance in tobacco and rice. These results indicate that *HSP* genes from various crops play a key role in developing thermotolerance.

### Vegetables

Vegetable crops are very susceptible to abiotic stresses such as high and low temperatures. Therefore, the development of varieties that are tolerant to heat and cold stresses is an important goal for improvement in crop productivity. Recently investigators have examined the protective roles of HSP and HSF against heat and cold stresses in transgenic vegetables. Li et al. (2003) reported that increased activity of soluble isoforms of ascorbate peroxidase (APX) and tolerance were observed in the transgenic tomato plants overexpressing *AtHSFA1b-gusA* fusion gene under heat and cold stress conditions. In addition, 15 heat tolerant tomato lines were isolated through screening of over 4000 ethyl methanesulfonate (EMS) Micro-Tom mutants. Among the selected heat tolerant mutants, the HT7 line displayed much higher fruit number and total pollen number with enhanced viability under HS conditions. Higher expression levels of *SIHSFA1b3*, which is known as a master regulator that activates HSR (Mishra et al., 2002), and *HSP101* were detected in the leaves of HT7 compared to those of WT after long-term exposure to HS, suggesting that HT7 could be used as a breeding material for production of tomato with improved heat tolerance (Pham et al., 2020). Also, up and downregulated expression of *HSP23*.*8* made it possible for each transgenic plant to display the opposite phenotype under low temperature conditions: Transgenic plants overexpressing *HSP23.8* gene showed increased cold tolerance whereas decreased chilling tolerance, wilting, skin wrinkles and partial discoloration were observed in the transgenic plant with reduced expression of *HSP23.8* gene (Escobar et al., 2021). Similar studies have reported that the *HSP17.7* gene plays a role in the HS tolerance in potato (Ahn and Zimmerman, 2006) and carrot (Malik et al., 1999). Recently, it has been reported that HS tolerance decreases in pepper when the *CaHSP60-6* gene is down-regulated by virus-induced gene silencing (VIGS) (Haq et al., 2019). In particular, CRISPR-Cas9 based gene knockout was applied to *GmHSP90A2* in soybean, and the *GmHSP90A2* mutant exhibited reduced heat tolerance (Huang et al., 2019). In conclusion, major *HSP* and *HSF* genes are tightly related to thermotolerance of vegetables. Thus, continuous efforts to identify detailed functions and working mechanisms of *HSP* and *HSF* genes are needed for the generation of vegetables with enhanced heat/cold tolerance traits through precise manipulation of genetic elements.

## Conclusion and Future Prospects

Climate change including global warming is causing abrupt changes in weather patterns, and extreme weather events that threaten crop yields. Elevated temperatures, in particular, will have a severe influence on the productivity and yields of vegetables in agricultural fields. It is, therefore, indispensible to understand the sophisticated mechanisms vegetable crops use to adapt to changing temperature environments, from the signal perception to gene expression in reponse to HS.

As mentioned above, recent research has elucidated that an interplay of cooperative HSP, HSF, and HSR mechanisms orchestrate the expression of heat-responsive genes as the plant response to HS. Furthermore, research identifying TFs related to abiotic stresses and their molecular functions has contributed to the expansion of knowledge for the production of crops with desired traits through genetic manipulation and/or molecular breeding. Functional and cellular roles of some key TFs such as HSFA1s and DREB2A have been determined in transcriptional networks of HSR at the post-translational levels during HS. Nevertheless, the current information on the functional roles of *HSP* and *HSF* genes in vegetable crops is still insufficient for their practical application to breeding. Transcriptional regulation between HSPs and HSFs, and in-depth working mechanisms and pathways of heat-related proteins during HSR remain to be explored.

Chromatin immunoprecipitation sequencing (ChIP-seq) for protein-protein complexes and reverse ChIP for mining the upstream-gene regulatory sequences have been shown to be effective tools to investigate potential interaction networks between regulatory regions in HSE and proteins, respectively (Machanick and Bailey, 2011; Shim et al., 2021). It will be necessary to utilize these techniques to clarify the in-depth mechanism underlying the gene regulatory relationships in the HSPs and HSFs of vegetable crops during HSR. It is becoming evident that microRNAs, small RNAs, and epigenetic modulations in DNA, RNA, and protein species play a pivotal role in HS memory (Guan et al., 2013; Stief et al., 2014a,b; Lämke et al., 2016). Advances in high-throughput small RNA sequences (RNA-seq) together with methylated DNA and RNA-sequencing combined with IP will be of help in determining the functions of TFs and epigenetic regulators (Pall and Hamilton, 2008; Zhang H. et al., 2018; Shen et al., 2019; Lee et al., 2021). In addition, state-of-art next-generation sequencing (NGS) including quantitative trait loci (QTL)-sequencing, genotyping-by-sequencing (GBS), and genome-wide association studies (GWAS) have been successfully developed and adopted for deciphering comprehensive genome sequences, thus facilitating the identification of a wide variety of molecular markers corresponding to target traits in crops (Han et al., 2016; Jo et al., 2017; Lee et al., 2020; Jha et al., 2021). Candidate and/or identified genes crucial for thermotolerant-traits and HS-related pathways can be used for production of transgenic vegetable crops via genetic engineering. Furthermore, the clustered regularly interspaced short palindromic repeats (CRISPR)/CRISPR-associated protein 9 (Cas9) and dead Cas9 (dCas9) systems have been extensively introduced into crop biotechnology as powerful tools for gene/genome editing in spite of controversial GMO and non-GMO issues (Liu D. et al., 2016; Pramanik et al., 2020; Gao, 2021; Kim et al., 2021). Indeed, “Sicilian Rouge High GABA tomato” was recently developed by using the CRISPR/Cas9 gene editing technology. It contains high levels of gamma-aminobutyric acid (GABA), an amino acid believed to aid relaxation and help lower blood pressure.^2^ All the aforementioned technologies can be utilized for dissecting action modes and intricate networks of HSP, HSF and HSR for thormotolerance in vegetable crops.

## Author Contributions

All authors listed have made a substantial, direct and intellectual contribution to the work, and approved it for publication.

## Conflict of Interest

The authors declare that the research was conducted in the absence of any commercial or financial relationships that could be construed as a potential conflict of interest.

## Publisher’s Note

All claims expressed in this article are solely those of the authors and do not necessarily represent those of their affiliated organizations, or those of the publisher, the editors and the reviewers. Any product that may be evaluated in this article, or claim that may be made by its manufacturer, is not guaranteed or endorsed by the publisher.

## Footnotes

1
www.ipcc.ch/

2
https://the-japan-news.com/news/article/0007780624
